# The COVID-19 Gene and Drug Set Library

**DOI:** 10.21203/rs.3.rs-28582/v1

**Published:** 2020-05-13

**Authors:** Maxim V. Kuleshov, Daniel J.B. Clarke, Eryk Kropiwnicki, Kathleen M. Jagodnik, Alon Bartal, John E. Evangelista, Abigail Zhou, Laura B. Ferguson, Alexander Lachmann, Avi Ma’ayan

**Affiliations:** 1 Department of Pharmacological Sciences; Mount Sinai Center for Bioinformatics; Big Data to Knowledge, Library of Integrated Network-based Cellular Signatures, Data Coordination and Integration Center (BD2K-LINCS DCIC); Knowledge Management Center for Illuminating the Druggable Genome (KMC-IDG); Icahn School of Medicine at Mount Sinai, One Gustave L. Levy Place, Box 1603, New York, NY 10029, USA; 2 Department of Neurology; Dell Medical School; University of Texas at Austin; 1601 Trinity Street, Bldg B, Austin, TX 78712, USA.

## Abstract

The coronavirus (CoV) severe acute respiratory syndrome (SARS)-CoV-2 (COVID-19) pandemic has received rapid response by the research community to offer suggestions for repurposing of approved drugs as well as to improve our understanding of the COVID-19 viral life cycle molecular mechanisms. In a short period, tens of thousands of research preprints and other publications have emerged including those that report lists of experimentally validated drugs and compounds as potential COVID-19 therapies. In addition, gene sets from interacting COVID-19 virus-host proteins and differentially expressed genes when comparing infected to uninfected cells are being published at a fast rate. To organize this rapidly accumulating knowledge, we developed the COVID-19 Gene and Drug Set Library (https://amp.pharm.mssm.edu/covid19/), a collection of gene and drug sets related to COVID-19 research from multiple sources. The COVID-19 Gene and Drug Set Library is delivered as a web-based interface that enables users to view, download, analyze, visualize, and contribute gene and drug sets related to COVID-19 research. To evaluate the content of the library, we performed several analyses including comparing the results from 6 in-vitro drug screens for COVID-19 repurposing candidates. Surprisingly, we observe little overlap across these initial screens. The most common and unique hit across these screen is mefloquine, a malaria drug that should receive more attention as a potential therapeutic for COVID-19. Overall, the library of gene and drug sets can be used to identify community consensus, make researchers and clinicians aware of the development of new potential therapies, as well as allow the research community to work together towards a cure for COVID-19.

## Introduction

Severe acute respiratory syndrome coronavirus 2 (SARS-CoV-2) is a novel coronavirus that was first detected in Wuhan, Hubei Province, China in November 2019. Infection with SARS-CoV-2 causes the coronavirus disease (COVID-19). Globally, there are more than 2.9 million confirmed COVID-19 cases and 203,000 reported deaths (as of April 25, 2020). The World Health Organization declared COVID-19 a pandemic on March 11, 2020. Many biomedical researchers have been shifting their efforts to battle the coronavirus COVID-19 pandemic. One area of activity is computationally prioritizing and experimentally testing approved drugs for repurposing as candidate therapies for attenuating COVID-19 infection. Drug repurposing studies present a promising avenue for quickly offering a treatment for COVID-19 because these drugs have known safety profiles. So far, drug repurposing studies can be categorized into three groups: in-vitro screens (1–6), computational predictions based on structural biology methods (7–9), and computational predictions based on network models and transcriptomics (10–12). Few studies have validated top computational predictions in cell-based models for COVID-19 cell based models (7,10,11). The lists of drugs mentioned in these studies can be analyzed for consensus, and suggested drugs can be grouped by their type. At the same time, many researchers attempt to understand the molecular mechanisms of the COVID-19 virus life cycle. Much attention has been given to a study that profiled, with mass-spectrometry proteomics, host proteins that interact with each of the COVID-19 proteins (11). Another important dataset produced RNA-seq gene expression signatures from various relevant human cell lines, ferret model lungs, and human lung biopsies before and after COVID-19 infection (13). These are just two examples of the many studies that produce gene sets that can be organized and compared. In the past, we developed a crowdsourcing project where we asked the community to identify gene expression signatures from drug, gene, and disease perturbations (14). The collection of over 6,000 signatures that we collected with the help of >70 contributors from around the world enabled us to produce a useful database called CREEDS. Similarly, for this project, we developed a crowdsourcing project to integrate gene and drug sets related to COVID-19 research collected with the assistance of the research community.

## Methods

### Collecting COVID-19 Drug Sets from Drug Repurposing Publications

Since the emergence of the COVID-19 epidemic, tens of thousands of new publications related to COVID-19 research have emerged in a very short period (2 months). We continually survey these publications to identify research that describes drug repurposing efforts, and manually extract drug sets from these studies to populate the drug set library. We also submit to the platform published drug sets from historical sources such as those from studies that listed drugs showing antiviral activity for other related viruses. So far, we have collected 20 drug repurposing publications (Table 1). An updated version of this table is maintained here:

https://docs.qooqle.eom/spreadsheets/d/1×6aKaZGadfLqNrQoFQwLlRhCXfUGYiRbzwYipIon_WM/edit?usp=sharing

To assist us with developing and maintaining the collection, we have received help from the research community by allowing researchers to upload gene and drug sets to the database. These submissions are manually evaluated before making them publicly available.

### Collecting SARS Signatures from GEO with GEO2Enrichr and GEN3VA

A set of 35 gene expression signatures resulting from infections by different coronaviruses for different cell types and tissues, with expression data originating from the gene expression omnibus (GEO) database, was processed using the GEO2Enrichr tool (15) and stored on the GEN3VA platform (16). The 70 entries were submitted to the COVID-19 crowdsourcing platform, with an upregulated and a downregulated gene set associated with each signature. The GEN3VA report for these signatures is available here:

https://amp.pharm.mssm.edu/gen3va/report/646/SARS.

### Collecting COVID-19-Related Gene Sets with Geneshot

Geneshot (17) is a platform that we developed to convert PubMed searches into gene sets. Using Geneshot, gene sets associated with the search terms SARS, SARS-CoV, MERS-CoV, ACE2, and TMPRSS2 were created using both the AutoRIF and GeneRIF (18) methods. Additionally, top COVID-19 drug repurposing candidates reported in recent literature (Table 1), including chloroquine and hydroxychloroquine, were included. Predictions of additional genes potentially associated with these terms were also added to the COVID-19 gene set library. These predictions were based on the literature-associated genes using each of five strategies: Co-occurrence via AutoRIF, GeneRIF, Enrichr (19), Tagger (20), and co-expression using data from ARCHS4 (21).

### Collecting COVID-19 Drug Sets from Twitter

Twitter is an important source for timely discussions related to therapeutics for COVID-19, including drug repurposing efforts and clinical trials. Using the Twitter API, we query Twitter daily with a list of more than 14,000 drug terms and their synonyms to collect tweets that mentioned these drugs in context of COVID-19. The drug search list was curated from DrugBank (22), L1000FWD (23), and the list of drugs submitted to the COVID19 drug and gene set library website. We then filter the identified tweets for those that are co-mentioned with COVID-19, and SARS linguistic variations. For each drug, we counted the occurrences of tweets and recorded a tally of mentions for each day. Data collection continues with daily reports, tweet IDs of the tweets originating the discussions, and the longitudinal drug trends. These data are shared publicly on GitHub:https://github.com/MaayanLab/COVID19DrugsTrendTracker/tree/master/daily_reports

Each day the set of discussed drugs on Twitter are automatically deposited into the COVID-19 drug set library via an API. This approach enables real time trend detection of the the most discussed drugs as potential therapeutics for COVID-19 while enriching the content of the COVID-19 drug and gene set library.

### Developing the COVID-19 Gene and Drug Set Library Website

**Fig. 1:**
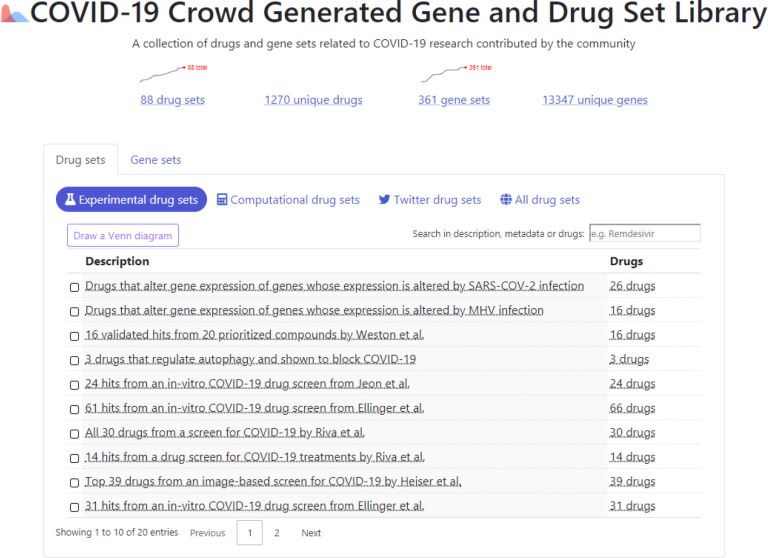
Screenshot from the landing page of the COVID-19 Drug and Gene Set Library

The COVID-19 gene and drug set library website has five sortable and searchable tables that list the drug and gene sets (Fig. 1). Sorting can be based on the date of submission, alphabetical ordering, or list size. The two tables are searchable via metadata terms such as title, authors, and descriptions, as well as via data search for specific gene or drug names. Users can download each gene set or drug set as well as the entire library. In addition, each gene set is provided with the option to perform gene set enrichment analysis with Enrichr (19), while genes are linked to Harmonizome (24) for further interrogation. The individual drugs that map to known compounds are linkable to their corresponding DrugBank landing pages (22). The website enables users to submit drug and gene sets related to COVID-19 research by completing a simple form. The form includes a dataset title, a URL source, and a description that explains how the set is relevant to COVID-19 research. The submitter is also provided with mechanisms to add additional metadata terms that can describe the cell type, tissue, organism, and other critical information about the submitted set. Users can specify the category of metadata provided, allowing for a broad set of additional metadata about each set. Users can also opt to submit their contact information; this information is kept private, but users can opt-in to make it public. Once a user submits a contribution to the site, their dataset is directed to a review queue in which we can examine the validity and relevance of the contribution. The reviewing process enables an administrator to approve or reject the submitted set. If approved, the set is added to the library. To make it easy for contributors to submit multiple sets, users can access the site via API. The code behind the site is open source and available at: https://github.com/MaayanLab/covid19_crowd_library

### Expression Analysis of In-Vitro Screens Hits

Drug sets extracted from 3 in-vitro screens (1–3) were first identified. The drugs were matched to drugs profiled by the L1000 assay available from GSE92742. Average signatures for each drug were computed by taking the z-score mean for each gene. Clusters were identified based on the average signatures using hierarchical clustering. Differential z-scores of genes relative to the two clusters were identified using the t-test statistic. The top up and down differentially expressed genes in each cluster were submitted to Enrichr for gene set enrichment analysis. To quantify the z-scores of genes co-expressed with ACE2, we calculated the correlation over 2,000 randomly sampled drug signatures from the L1000 database. We then calculated the mean z-scores of the top 50 correlated genes to ACE2 and compared those values against a distribution calculated from sampling 50 random genes, repeatedly 10,000 times. The p-values were calculated against the sampled distribution and corrected for multiple hypothesis testing by applying the Bonferroni correction method. The code behind this analysis is open source and available at: https://github.com/maayanlab/covid19l1000

## Results

### Analysis and Visualization of Consensus Drug and Gene Sets

**Fig. 3:**
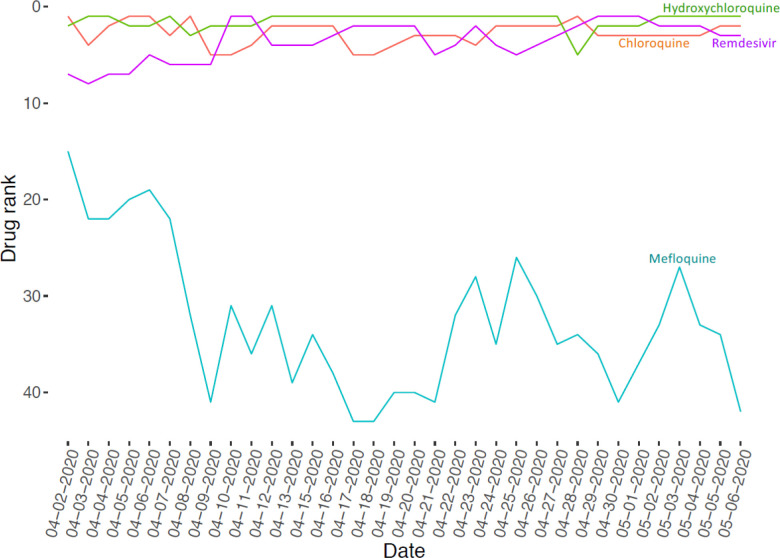
Ranks of drugs based on their mentions on Twitter in context of COVID-19 over time.

So far, we have collected 87 drug sets composed of 1265 unique drugs, and 361 gene sets consisting of 13,347 unique genes. The drug sets are subdivided into four categories: experimental (20), computational (31), Twitter (31), and other (5). The top 20 most frequent drugs and genes across all sets are displayed in Fig. 2A–E. The list of experimental drugs with most supportive evidence are hydroxychloroquine, mefloquine, chloroquine, and remdesivir (Fig. 2A). While hydroxychloroquine, chloroquine, and remdesivir are the most discussed drugs on Twitter, mefloquine received so far less attention (Fig. 3). While mefloquine is consistently ranked within the top 100 drugs each day, the anti-malaria drug mefloquine (25) is much less discussed and as such may be a good candidate for further experimental investigation (Fig. 3).

**Fig. 4:**
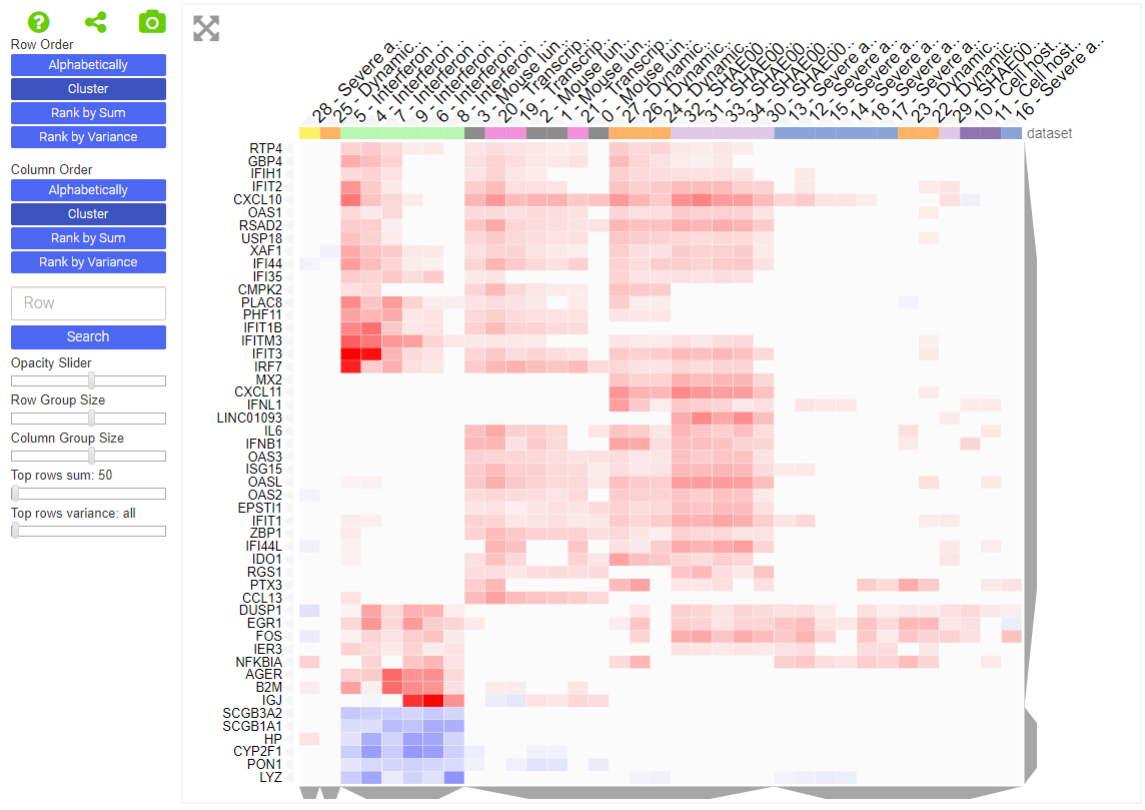
The SARS GEN3VA report gene view. The heatmap displays the most consistent up and down-regulated genes from 35 signature created from microarray studies where mammalian cells and tissues were challenged with SARS. The GEN3VA report is available from here: http://amp.pharm.mssm.edu/gen3va/report/646/SARS

**Fig. 5:**
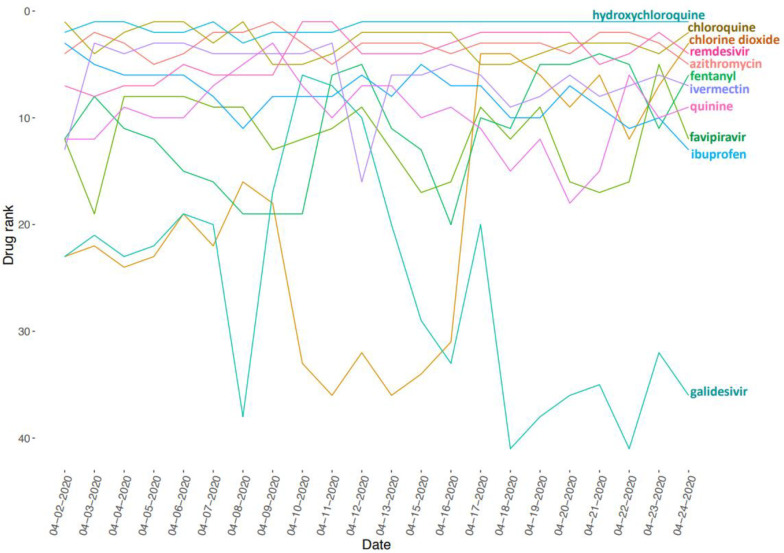
Ranks of drugs based on their mentions on Twitter in context of COVID-19 over time.

The top 20 most frequently submitted genes are all members of the innate immune response (Fig. 2E). These genes include the typical interferon and cytokine response genes observed to be involved in the response of human cells to most pathogens. These genes are also listed as the top differentially expressed upregulated genes from the GEN3VA report of 35 signatures (Fig. 4). However, the list here also includes the immediate early gene (IEG) module composed of the transcription factors EGR1 and FOS and the phosphatase DUSP1. Based on enrichment analysis with Enrichr (19), the top 10 downregulated genes from the GEN3VA report include five genes (SH3BGRL, LGALS1, COX7A2, CRIP1 and LYZ) that are highly expressed in dendritic cells (p-value < 0.003, Fisher exact test), suggesting that there may be a depletion of dendritic cells due to SARS infections. Our ability to collect tweets about drugs using the Twitter API enables us to track trends about new drugs that are increasingly discussed on this social media platform. During the period between April, 2nd to April 24th, 2020, we noticed the rise and fall of discussions about the drug galidesivir (Fig. 5). Galidesivir (BCX4430) is an adenosine analog that was previously suggested as a potential antiviral drug for several related viral diseases (26–28).

**Fig. 6:**
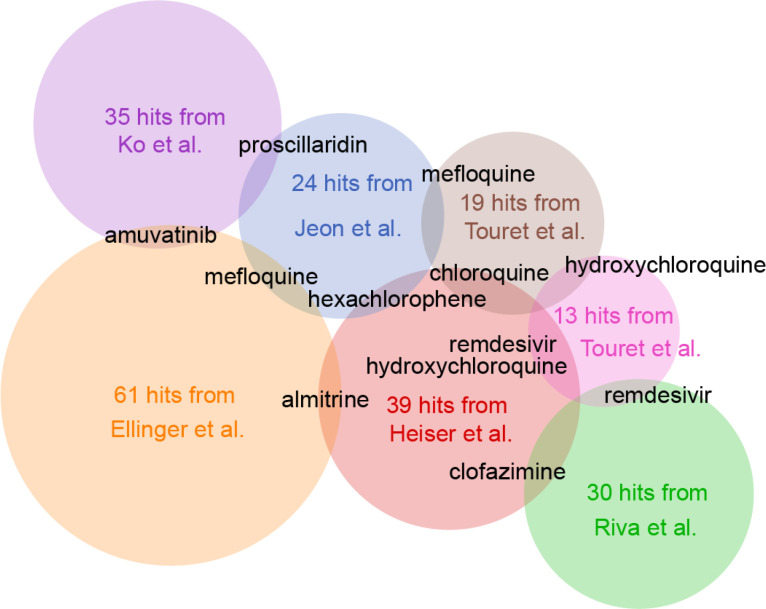
Overlap analysis across six in-vitro drug repurposing screens for COVID-19 inhibitors

While most of the drug sets in the library are from studies that utilized computational methods to predict drugs, few are from large-scale approved drug screens (1–6). Using the Venn diagram tool developed for ad-hoc analysis of the sets in the library, we compared the results from six *in-vitro* COVID-19 drug screen studies (Fig. 6). Although, there is very little overlap across these drug screens, where only one or two drugs are shared across these experimental studies, some interesting less discussed hits emerge. Namely, these are amuvatinib, proscillaridin, mefloquine, hexachlorophene, clofazimine, and almitrine. Amuvatinib is a multi-targeted tyrosine kinase inhibitor (29) and proscillaridin is an organic compound that is used an old cardiotonic steroid but more recently suggested as a cancer agent (30). Hexachlorophene is a disinfectant that is used in dermatological products (31). Clofazimine is a drug used to treat leprosy and its mechanisms of action suggest that it interferes with DNA synthesis (32). Almitrine is a drug that stimulates respiratory respiration by activating receptors of carotid bodies (33). It is used in the treatment of chronic obstructive pulmonary disease (34), and as such it is most relevant to the COVID-19 symptoms.

### ACE2 Up- or Down-Regulation Effects of Drug Hits?

**Fig. 7:**
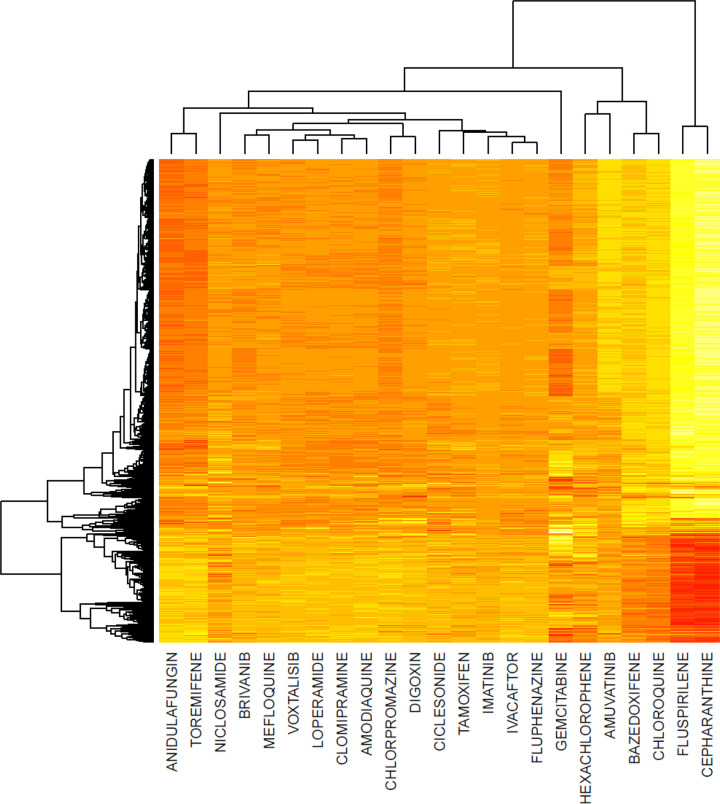
Hierarchical clustering of gene expression signatures for 23 drug hits from 3 published in-vitro screens that also have L1000 profiling gene expression data. The rows represent genes where red represents high expression and yellow low expression.

To further explore the molecular effects of positive hits from the in-vitro drug screens, and to demonstrate the utility of the collected library, we developed a case study that asks whether drugs that have been shown to inhibit COVID-19 infection of human cells in-vitro, up- or down-regulate the ACE2 gene. ACE2 is the suspected cell surface receptor for COVID-19 (35), and cells that do not express this gene have been shown to be less prone to COVID-19 infection. Since it is still undetermined whether it is desired to up- or down-regulate ACE2 expression, we queried each drug hit from two published in-vitro drugs screens against the LINCS L1000 data (36). We identified 23 drug hits from the screens that have been profiled by L1000 comprising a total of 1251 differential gene expression profiles. 17 of these drugs display, on average, an increase in ACE2 expression, while 6 drugs display a decrease in expression of ACE2. Overall, the gene expression signatures for these 23 drugs can be categorized into two distinct clusters (Fig. 7). The observed difference in gene expression signatures suggest different modes of action for these drugs. The drugs that are most similar to chloroquine exhibit consistent up-regulation of genes highly correlated to ACE2. Chloroquine gene expression signatures on average up-regulate the top 50 most correlated genes to ACE2 (p=2.55e-03). In addition, cepharanthine, fluspirilene, bazedoxifene, and amuvatinib also up-regulate the same set of ACE2 most correlated genes (p=3.17e-17, p=7.01e-13, p=2.74e-03, and p=4.94e-03, respectively). These 50 genes are available in Table 2. The strong dysregulation of ACE2 associated genes could point to a similar mode of action regarding antiviral activity. Enrichment analysis of the consensus genes upregulated by the drugs that co-cluster with chloroquine dislpay up regulation of immune response genes and down-regulation of cell cycle genes. This suggests that the drugs in the chloroquine cluster are pro-inflammatory while the other cluster of drugs contains anti-inflammatory drugs.

Up genes in the chloroquine cluster:

https://amp.pharm.mssm.edu/Enrichr/enrich?dataset=d9201bbba4dff47598bcf66f3db3e93e

Down genes in the chloroquine cluster:

https://amp.pharm.mssm.edu/Enrichr/enrich?dataset=698f9a31fbc85e13da57bce68a543083

## Discussion

Here we describe a platform created to collect drug and gene sets related to COVID-19 research using various methods of data accrual. All of the top 10 genes associated with COVID-19 are interferon-related (ISG15, IRF7, OAS1, IFITM3, MX1, IFIH1, STAT1, IFIT3, and EIF2AK2). This is consistent with our knowledge that type I (IFN-α, IFN-β) and type III interferon (IFN-λ) systems are the primary defense against viral infections. It has been hypothesized that hyper-inflammation in COVID-19 could drive disease severity and would be amenable to treatment with drugs that reduce inflammation (37). However, this remains controversial as the high level of antiviral response could be reflective of increased viral burden rather than an inappropriate host response (38). The most striking result from the meta-analysis applied to the content of the library is the little overlap across drug screen studies. It is expected that experimental validation of drugs to inhibit COVID-19 in-vitro will be more consistent. The inconsistency across these studies could be due to a need to produce results quickly due to the urgency for discovering potential treatments. Regardless, there is some overlap, and there is expectation that more screens will be published and top leads will advance to animal models and human trials for further testing. To prioritize compounds that may treat COVID-19, some researchers have used the strategy of finding drugs that modulate genes related to ACE2 gene expression (39). We found a highly significant up-regulation of the genes most correlated with ACE2 by chloroquine and related compounds in the L1000 database. This finding suggests that identifying compounds that up-regulate genes correlated with ACE2 expression could be a useful approach for identifying more compounds that inhibit COVID-19 in mammalian cells. However, it should be noted that other considerations must be taken into account, including the known side effects for the compounds and whether dosing would allow for high enough drug concentration to achieve therapeutic effects.

It should be clear that the consensus analysis results should be viewed with caution. The most common drugs are not necessarily the most efficacious or promising treatments. At the same time, the most common genes may not be the most relevant to understand COVID-19 research. It should be noted that not all drug sets and gene sets have equal weight in quality and relevancy. A list of computationally predicted drugs is not as useful towards identifying a therapy for COVID-19 when compared with a list of experimentally validated drugs. A list of upregulated genes after COVID-19 infection of cells may provide more useful information about the virus life cycle when compared with a list of genes returned from a Geneshot search using the term SARS. Hence, the users of the data collected into the library should be aware of such limitations. With these limitations in mind, we hope that researchers will be able to develop or refine hypotheses from the snapshot overview our platform provides, and then delve deeper into the studies most relevant to their questions. For example, the GEN3VA report revealed a down-regulation of genes known to be enriched in dendritic cells (DCs) after infection with SARS. SARS-CoV-1 is known to infect DCs and impair DC function (40).

In a period of rapid development of methods and data related to COVID-19 research, it is critical to provide means to organize the accumulated information in a way that it can be summarized and reused. The COVID-19 gene set and drug set library provides such utility. The library of drug and gene sets can be used to identify community consensus and make researchers and clinicians aware of the development of new potential therapies as they become available, as well as allow the research community to work together towards a cure for COVID-19. Social media has played a critical role in enabling scientists to communicate results rapidly and exchange ideas. By mining the trends about discussions on Twitter about drugs in context of COVID-19, we are able to keep track with global trends before these are reported in scientific journals.

## Figures and Tables

**Fig. 2A:**
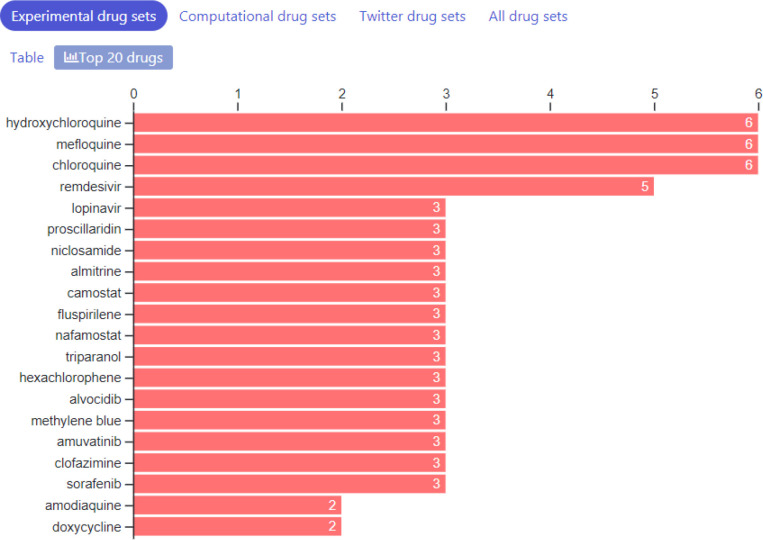
Counts of most common drugs from the collection of experimental studied that reported lists of drugs that inhibit COVID-19.

**Fig. 2B:**
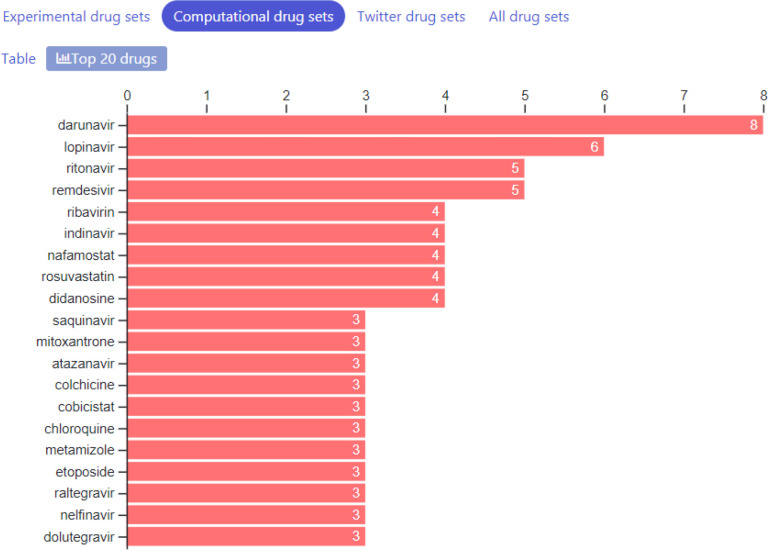
Counts of most common drugs from the collection of computational studied that reported lists of drugs that may inhibit COVID-19.

**Fig. 2C:**
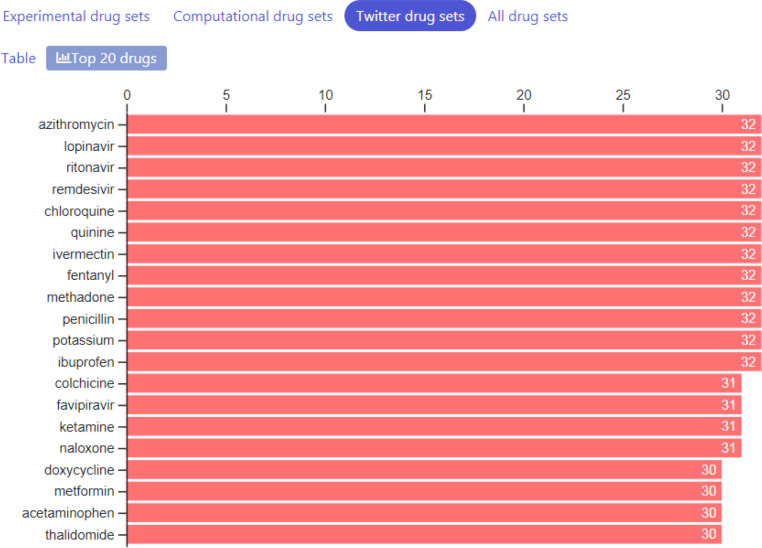
Counts of most common drugs discussed each day on Twitter during the period from April 4, 2020 to May 6, 2020.

**Fig. 2D:**
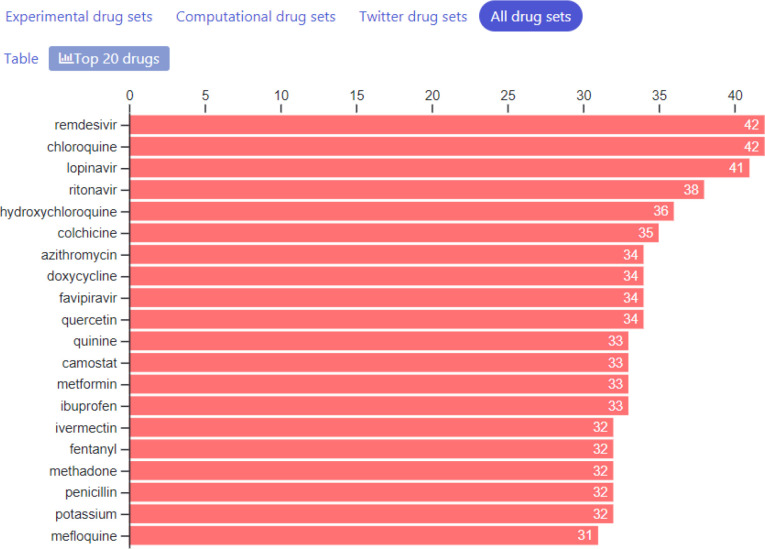
Counts of most common drugs from the collection of all drug sets in the library.

**Fig. 2E:**
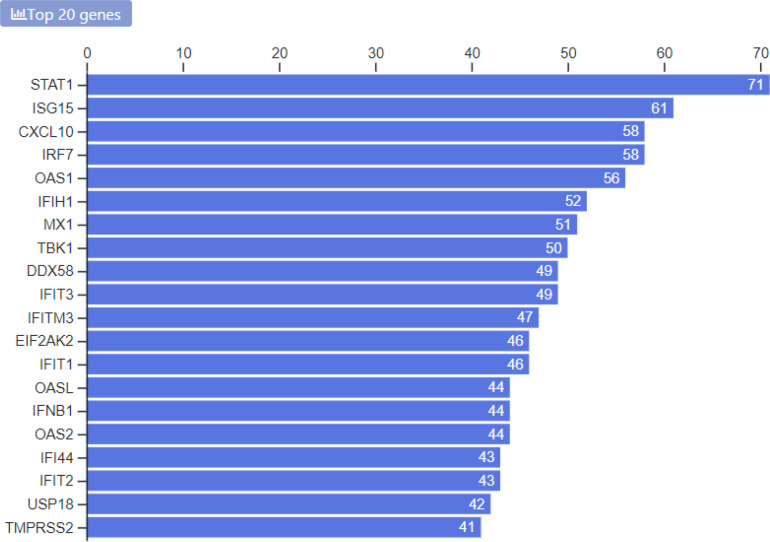
Counts of most common genes from the collection of all gene sets in the library.

**Table 1:** 

First author	Journal	Drugs	Exper.	Comp.	Method	Target	Cells
Jeon	biorxiv	24	Yes	No	Inhibition assay		Vero cells
Gordon	biorxiv	63	No	Yes	Mass-spec Docking	Multiple	HEK293T
Farag	chemrxiv	71	No	Yes	Docking	Mpro	
Wang	chemrxiv	21	No	Yes	Docking	Mpro	
Contini	chemrxiv	19	No	Yes	Docking	Mpro & C3Lpro	
Kumar	chemrxiv	10	No	Yes	Docking	Mpro	
Zhou	Cell Discovery	16	No	Yes	Network Analysis		
Aly	chemrxiv	7	No	Yes	Docking	Mpro	
Jin	Nature	7	Yes	Yes	Docking	Mpro	
Rensi	chemrxiv	21	No	Yes	Docking	TMPRSS2	
Xing	biorxiv	11	Yes	Yes	L1000 Inhibition assay		Vero cells
Touret	biorxiv	90	Yes	No	Inhibition assay		Vero cells
Ko	biorxiv	35	Yes	No	Inhibition assay		Vero cells
Nguyen	biorxiv	84	No	Yes	Docking	Mpro	
Ge	biorxiv	1	Yes	Yes	L1000 Network Analysis	PARP1	PBMCs
Alakwaa	mSystems	4	No	Yes	L1000 scRNA-seq		
Beck	Comput Struct Biotech J.	8	No	Yes	Docking	Multiple	
Chen	F1000Res.	15	No	Yes	Docking	C3Lpro	
Cava	MDPI	36	No	Yes	Network Analysis		
Ellinger	Research Square	64	Yes	No	Inhibition assay		Caco-2
Heiser	biorxiv	100	Yes	No	Image-based assay		HRCE cells
Riva	biorxiv	30	Yes	No	Inhibition assay		Vero cells

**Table 2:** 

SLC13A1, LRRC19, HAVCR1, HHLA2, CT62, LDHAL6B, SLC22A11, DDX4, CLCN5, SLC25A31, GIPC2, GUCY2C, ABCB1, TMPRSS15, RBMXL2, TM4SF20, KDM4D, SLCO4C1, PRM2, RNF32, CNTN6, ACSL6, UBQLN3, HNF4G, ANKRD7, MXD1, SLC17A3, IL12RB2, ANKRD40CL, SLC6A20, SLC5A12, SLC10A2, ACRV1, ALPI, EPPIN, IL17A, AKAP4, HKDC1, BRDT, TSBP1, SLC22A2, P2RY1, FOXD2, NAT8B, ASCL2, IL12A, CREB5, DMRT1, PHF14
